# *CASC11* and *PVT1* spliced transcripts play an oncogenic role in colorectal carcinogenesis

**DOI:** 10.3389/fonc.2022.954634

**Published:** 2022-08-16

**Authors:** Mina Zamani, Ali-Mohammad Foroughmand, Mohammad-Reza Hajjari, Babak Bakhshinejad, Rory Johnson, Hamid Galehdari

**Affiliations:** ^1^ Department of Biology, Faculty of Science, Shahid Chamran University of Ahvaz, Ahvaz, Iran; ^2^ Department of Genetics, Faculty of Biological Sciences, Tarbiat Modares University, Tehran, Iran; ^3^ Department of Medical Oncology, Inselspital, Bern University Hospital, University of Bern, Bern, Switzerland; ^4^ Department for BioMedical Research, University of Bern, Bern, Switzerland; ^5^ School of Biology and Environmental Science, University College Dublin, Dublin, Ireland; ^6^ Conway Institute for Biomolecular and Biomedical Research, University College Dublin, Dublin, Ireland

**Keywords:** colorectal cancer, 8q24, MYC, noncoding RNA, PVT1, CASC11

## Abstract

Cancer is fundamentally a genetic disorder that alters cellular information flow toward aberrant growth. The coding part accounts for less than 2% of the human genome, and it has become apparent that aberrations within the noncoding genome drive important cancer phenotypes. The numerous carcinogenesis-related genomic variations in the 8q24 region include single nucleotide variations (SNVs), copy number variations (CNVs), and viral integrations occur in the neighboring areas of the *MYC* locus. It seems that *MYC* is not the only target of these alterations. The *MYC*-proximal mutations may act *via* regulatory noncoding RNAs (ncRNAs). In this study, gene expression analyses indicated that the expression of some *PVT1* spliced linear transcripts, *CircPVT1*, *CASC11*, and *MYC* is increased in colorectal cancer (CRC). Moreover, the expression of these genes is associated with some clinicopathological characteristics of CRC. Also, *in vitro* studies in CRC cell lines demonstrated that *CASC11* is mostly detected in the nucleus, and different transcripts of *PVT1* have different preferences for nuclear and cytoplasmic parts. Furthermore, perturbation of *PVT1* expression and concomitant perturbation in *PVT1* and *CASC11* expression caused *MYC* overexpression. It seems that transcription of *MYC* is under regulatory control at the transcriptional level, i.e., initiation and elongation of transcription by its neighboring genes. Altogether, the current data provide evidence for the notion that these noncoding transcripts can significantly participate in the *MYC* regulation network and in the carcinogenesis of colorectal cells.

## Introduction

The human transcriptome includes over 90,000 expressed genes. More than 70% of these genes are categorized as long noncoding RNAs (lncRNAs) (1). Up to now, only a small fraction of the thousands of annotated lncRNAs have been cataloged with a putative function (2). LncRNAs perform a variety of biological activities such as regulating chromatin topology, the scaffolding of proteins and other RNAs, acting as protein and RNA decoys and stabilizers, regulating neighboring genes, and producing micropeptides. They carry out these functions *via* RNA–RNA, RNA–DNA, and RNA–protein interactions through both *cis* and *trans* mechanisms (2, 3). The lncRNA plasmacytoma variant translocation 1 (*PVT1*) is located in 8q24.21, a part of the gene desert region on the long arm of chromosome 8 (8q24). The region 8q24 hosts many variations, such as single nucleotide variations (SNVs), copy number variations (CNVs), translocations, and viral integrations associated with carcinogenesis of the breast, prostate, bladder, colon, lung, blood, ovary, and pancreas in different ethnicities (4–8). Understanding the functional effects of the aberrant genetic characteristics of 8q24 associated with carcinogenesis remains challenging due to the complexity of the region (9). The most well-known resident in this region is the proto-oncogene *MYC* and other loci within the region, most of which are noncoding transcripts, remain opaque. Over the past years, several studies have found that *PVT1* is overexpressed in human tumors, including cervical, renal, gastric, serous melanoma, prostate, and leukemia malignancies where it functions as an oncogene (10–16). Another lncRNA located in this region, cancer susceptibility 11 (*CASC11*), has been found to promote gastric, colorectal, and hepatocellular cancers (17–19). Limited information is available on the functional analysis of ncRNA residents of 8q24, particularly in relation to *MYC* regulation. This highlights the importance and necessity of further studies to obtain a more detailed understanding of the role of lncRNAs located in 8q24 and possible regulatory interactions between these lncRNAs and *MYC*. In the current study, we evaluated the expression and possible *cis* relevance between *MYC*, *PVT1*, and *CASC11* in colorectal cancer (CRC). We found that *MYC* transcription is partly under the control of its neighboring lncRNAs, *PVT1* and *CASC11*.

## Materials and methods

### Sample collection

A total of 40 colorectal and normal margin fresh frozen samples were obtained from the Institute of Cancer (Tehran, Iran). The present study was approved by the Ethics Committee of Shahid Chamran University of Ahvaz, Ahvaz, Iran (Ethics code: EE/99.3.02.65802/scu.ac.ir).

### Cell lines and culture conditions

HCT116 and LoVo (both human colorectal carcinoma) cell lines were obtained from Bern University. HCT116 and LoVo were cultured in Dulbecco’s modified Eagle’s medium (DMEM) and F12/DMEM (Sigma-Aldrich^®^, USA), respectively. The medium was supplemented with 10% fetal bovine serum (FBS; Gibco^TM^, USA), 100 U/ml of penicillin, and 100 mg/ml of streptomycin (Life Technologies, USA). All cells were grown at 37°C in a 5% carbon dioxide humidified atmosphere.

### RNA extraction, cDNA synthesis, and Q-PCR

Total RNA was extracted from tissue samples using TRIzol^®^ reagent (Life Technologies, USA) followed by DNaseI (Takara Bio Inc., Shiga, Japan) digestion. Complementary DNA (cDNA) was synthesized by Prime Script™ RT Reagent Kit (Takara Bio Inc., Shiga, Japan). The list of primers for all targeted genes is indicated in **Supplementary Table S1**. Real-time PCR was performed using the SYBR^®^ Premix Ex-Taq™ II (Takara Bio Inc., Shiga, Japan).

The RNA fraction was isolated from cells using the Quick-RNA Miniprep kit (ZYMO Research, USA). The isolated RNA was subjected to on-column DNase I treatment and clean-up using the manufacturer’s protocol. RNA was converted to cDNA using GoScript™ Reverse Transcriptase (Promega, USA), random hexamer, and oligo dT primers. The expression of each of the individual transcripts was quantified by qRT-PCR (Applied Biosystems^®^ 7500 Real-Time, USA) using the indicated primers (**Supplementary Table S1**) and GoTaq qPCR Master Mix (Promega, USA). The relative gene expression was calculated as 2^−ΔΔCt^ (20), using *ACTB* and *GAPDH* as endogenous control genes. This method is a relative quantification of gene expression; Δ*C_T_* is the difference in the threshold cycle between the target and reference genes.

### TA cloning and Sanger sequencing

To find frequently spliced transcripts of *PVT1* in gastric and colorectal samples, total cDNA was prepared using random hexamer and oligo dT primers. Subsequently, PCR was performed using primers for the start (exons 1 and 2) and end (exon 9) of the *PVT1* transcript (ENST00000513868.2, NR_003367). The amplicons were excised from the gel and purified using AccuPrep^®^ Gel Purification Kit (Bioneer, Korea). Purified PCR products were then cloned into a pTG19-T vector (Sinaclon, Iran) using T4 DNA ligase (Fermentas, USA) and transformed into DH5α-competent cells. Recombinant colonies were positively selected through resistance to ampicillin (Sigma-Aldrich^®^, USA). Colony PCR was performed using universal M13 primers to select different transcripts of *PVT1*. Sanger sequencing of PCR products was done by ABI Prism 3700 automated genetic analyzer (Narges Medical Genetics and Prenatal Diagnosis Laboratory, Ahvaz, Iran).

### Generation of dCas9/KRAB expressing stable cell lines

LoVo and HCT116 cells were transfected with the vector carrying SID4x-dCas9-KRAB (Addgene 48227, modified to express red fluorescent protein (RFP)) using Lipofectamine 2000 (Thermo Fisher Scientific, USA). All cell types were selected with blasticidin (8 μg/ml) (Gibco^TM^, Thermo Fisher, USA) for 10 days and selected for RFP-positive cells by fluorescence-activated cell sorting (FACS).

### sgRNA pair design and cloning

Single-guide RNA (sgRNA) pairs were designed using CRISPETA (21), CRISPR-ERA (22), and GPP (23–26), then cloned into the pDECKO backbone as described previously (21). DECKO employs a two-step cloning methodology. Cloning is based on the Gibson assembly method. Briefly, in step 1, multiple oligonucleotides were assembled with the backbone plasmid to create an intermediate plasmid. In step 2, a constant “Insert-2” fragment was inserted within the new sequence created in the previous step. Established cells expressing dCas9/KRAB were then transfected with recombinant DECKO, including guides. After 48 h of selection *via* puromycin (2 μg/ml) (Gibco^TM^, Thermo Fisher, USA), the following tests were done for specific times. All sgRNA positions and sequences are indicated in **Supplementary Table S2**.

### Transfection

Cells (70% confluent in six-well plates) were transfected using Lipofectamine 2000 (Thermo Fisher Scientific, USA) with 2.5 μg of pDECKO plasmid following the provider’s guidelines. After 6 h, the transfection medium was replaced with fresh complete DMEM for HCT116 and F-12K for LoVo cells (10% FBS, 1% l-glutamine, and 1% penicillin–streptomycin).

After 1 day, cells were selected with puromycin (2 µg/ml). After 5 days of puromycin selection, cells were trypsinized and resuspended in PBS. A total of 10,000 cells per sample were sorted. qRT-PCR was then used to assess the results of gene knockdown.

### Cell viability test

Cell viability was determined using the 3-(4,5-dimethylthiazol-2-yl)-2,5-diphenyl-2H-tetrazolium bromide (MTT) assay according to the manufacturer’s instructions (Sigma-Aldrich^®^, USA). Briefly, identical numbers of cancerous cells in a 100-μl medium containing 10% FBS were seeded in triplicate onto 96-well plates and incubated overnight. On the first day at time 0 (T0), 20 μl of 5 mg/ml MTT was added to each well and incubated for an extra 4 h, followed by the addition of 200 μl of dimethylsulfoxide. For other timepoints, the same procedure was done every 24 h (24, 48, 72, and 96 h) after cell seeding. The absorbance was determined at 570 nm by a 96-well plate reader (TECAN, Switzerland), which is proportional to cell viability. All values were compared with the matching controls. Cell viability was calculated as the percentage of viability of test against control cells.

### Cell fractionation

Cell fractionation was conducted according to the REAP method (27), with some changes. Briefly, cells were collected using trypsin (Sigma-Aldrich^®^, USA) and spun down, and the cell pellets were resuspended in 900 μl of ice-cold 0.1% NP40 in PBS and triturated five times using a p1000 micropipette. In total, 300 μl of the lysate was removed as “whole cell lysate” and incubated with 0.5 μl of ribonuclease inhibitor (Promega, USA). The remaining (600 μl) material was centrifuged for 10–15 s in 1.5 ml microcentrifuge tubes, and 300 μl of supernatant was removed as the “cytosolic fraction,” followed by the addition of 0.5 μl of ribonuclease inhibitor. After removing the remaining supernatant, the pellet was resuspended in 1 ml of ice-cold 0.1% NP40 in PBS, centrifuged as described above for 10–15 s and the supernatant was discarded. The pellet (~20 μl) was resuspended with 200 μl of ice-cold PBS, designated as “nuclear fraction,” and incubated with 0.5 μl of ribonuclease inhibitor. Nuclear fractions and whole-cell lysates that contained DNA were sonicated. *GAPDH* mRNA and *MALAT1* lncRNA were used as cytoplasmic and nuclear markers, respectively.

### Statistical analysis

All statistical analyses were performed using GraphPad Prism version 7 (GraphPad Software Inc., USA). The comparison between gene expression data of two tumor and normal margin samples in two groups was done using the Wilcoxon test. Spearman’s rank correlation coefficient was used for correlation analysis of relative gene expression with clinical parameters and correlation analysis of gene expression data. ROC curves were generated for gene expression datasets of tumor and normal margin samples as patient and control values using the Wilson/Brown method.

## Results

### *PVT1* is alternatively spliced in colorectal cancer cells

Alternative splicing of lncRNAs is important in the regulation of a variety of human diseases, including cancer (28, 29). These genes are capable of producing multiple splice variants that can have distinct molecular functions through altering scaffold properties of lncRNAs, creating new ORFs for small peptides and disrupting an ORF from the main lncRNA, and producing various circular RNAs (circRNAs) (30).

**Figure 1:**
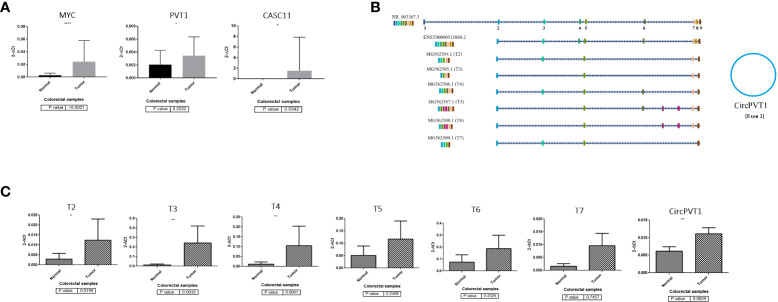
*PVT1*, *CASC11*, and *MYC* expression analysis in colorectal cancer. **(A)** *PVT1*, *CASC11*, and *MYC* are overexpressed significantly (*p* < 0.05) in CRC tumor samples. **(B)** *PVT1* transcripts and **(C)** their expression analysis in colorectal tumor samples. Most underrepresented transcripts are overexpressed in colorectal tumors compared to normal margin samples *P<0.05, **P<0.01, ****P<0.0001.

*PVT1* codes for linear ncRNA isoforms, circRNA, and microRNAs. Given the high number of alternatively spliced transcripts of the *PVT1* locus, we tried to trap various frequent linear transcripts of *PVT1* in our samples. We found six alternative transcripts of *PVT1* in the pooled RNA samples of colorectal tumor and normal tissue samples. These transcripts were registered in the NCBI database with the following accession numbers; MG562504.1 (T2), MG562505.1 (T3), MG562506.1 (T4), MG562507.1 (T5), MG562508.1 (T6), and MG562509.1 (T7) (**Figure 1C**).

### *MYC, PVT1*, and *CASC11* are overexpressed significantly in CRC tumors

To elucidate the role of *PVT1*, *CASC11*, and *MYC* in *CRC*, we assessed the expression levels of these genes using qRT-PCR for cancerous and adjacent normal tissues from colorectal cancer patients (**Figure 1**). Our data demonstrated that *PVT1* circular RNA (*CircPVT1*), *MYC*, and *CASC11* are significantly (*p* < 0.05) overexpressed in CRC tumor samples compared to normal margin tissues (**Figure 1A**). The relative expression of detected *PVT1* alternative transcripts was studied. A significant relative overexpression was observed for T2, T3, and T4 in CRC tumors compared to margin-normal samples (**Figures 1B, C**). The correlation analysis of gene expression in tumor to normal group ratio showed a moderate positive expression correlation between transcripts including T3/*CASC11* (*R* = 0.54, *p* = 0.003) and T5/T7 (*R* = 0.54, *p* = 0.003), indicating a possible coregulation between these transcripts and splicing from the same longer transcripts (**Supplementary Table S1**).

**Figure 2:**
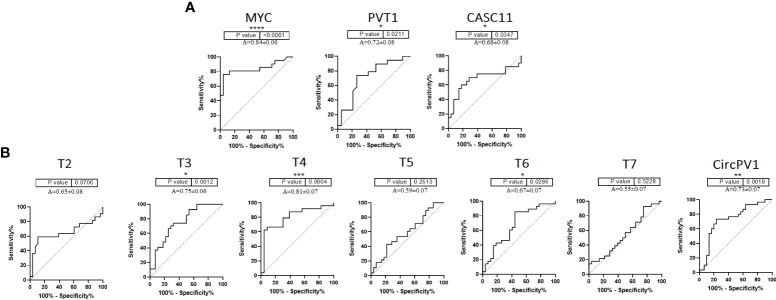
ROC curve of *PVT1*, *CASC11*, and *MYC* expression in colorectal tumors. **(A)** *PVT1*, *CASC11*, and *MYC* might be considered biomarkers for the early prognosis of colorectal cancer. **(B)** According to the ROC curves of *PVT1* transcripts in colorectal tumors, *T3*, *T4, T6, and CircPVT1* might be useful in the early detection of malignancy *P<0.05, **P<0.01, ***P<0.001, ****P<0.0001.

To further clarify the role of underrepresented genes in the pathogenesis of CRC, we performed a correlation analysis between gene expression data and clinicopathological features of tumors. In the CRC tissue samples, *MYC* expression was moderately correlated with tumor size (*R* = 0.43, *p* = 0.03) and pathological T (*R* = 0.60, *p* = 0.002). T5 was significantly correlated with family history (*R* = 0.39, *p* = 0.04) (**Supplementary Table S3**; **Supplementary Figure S1**). Next, ROC curves were generated to independently characterize the predictive value of underrepresented transcripts. The results of the ROC curve analyses (**Figure 2**) indicated that *MYC*, T4, *CircPVT1*, and T6 significantly exhibit AUC above 0.67 in CRC tissues.

### *MYC* expression is increased following *PVT1* perturbation

**Figure 3:**
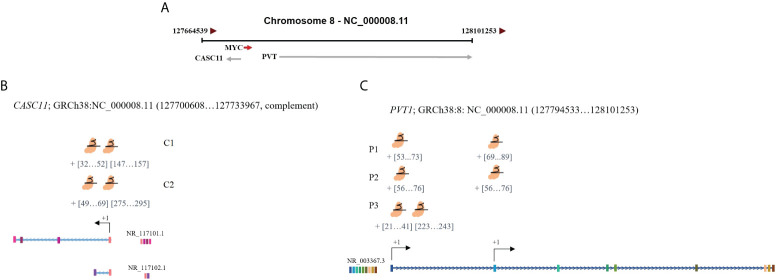
Schematic representation of the target positions of designed guides for *PVT1* and *CASC11*. **(A)** *PVT1* and *CASC11* are located downstream and upstream of *MYC*. **(B)** *CASC11*-sgRNAs and **(C)** *PVT1*-sgRNA target sites are near TSS. TSS, transcription start site.

**Figure 4:**
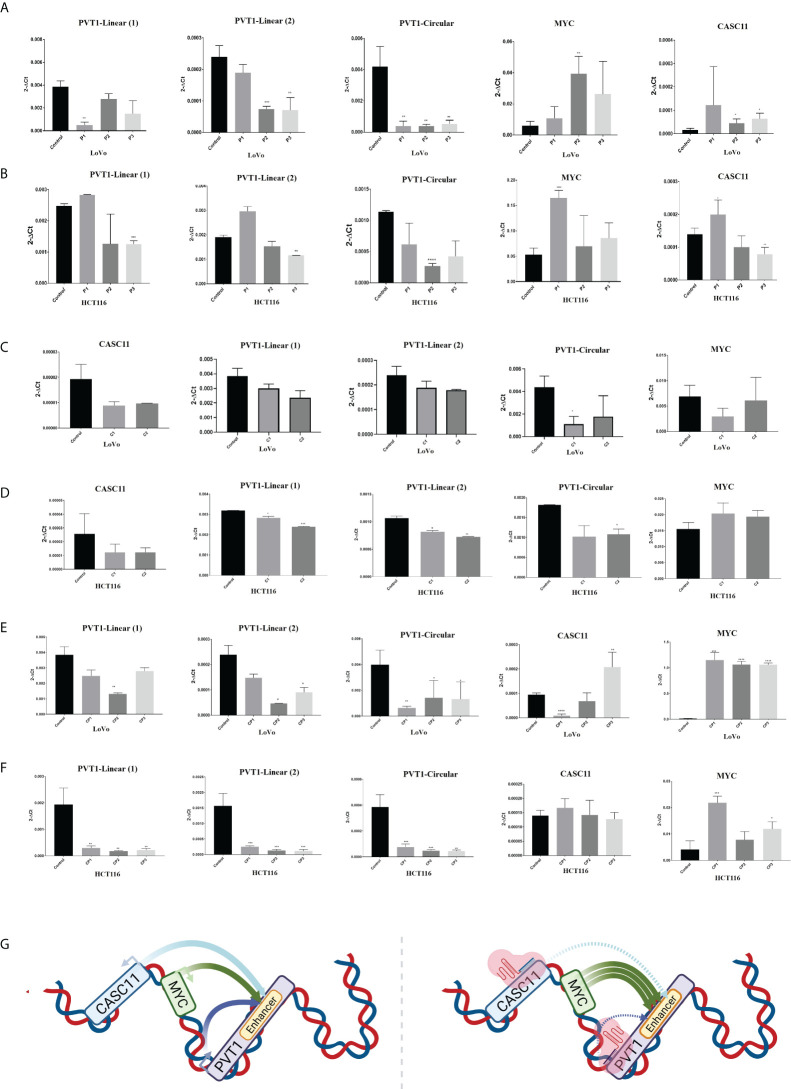
*PVT1* and *CASC11* expression perturbation in LoVo **(A)** and HCT116 **(B)** cells. P1: *PVT1* sgRNA pair 1, P2: *PVT1* sgRNA pair 2, P3: *PVT1* sgRNA pair 3, and control: non-targeting sgRNA. PVT1 expression was decreased by using P2 and P3 pair guides. *MYC* expression was increased after interference with *PVT1* expression. Perturbation of *CASC11* expression in **(C)** LoVo and **(D)** HCT116 cells. C1:*CASC11* sgRNA pair 1, C2: *CASC11* sgRNA pair 2, and control: non-targeting gRNA. *CASC11* expression was decreased by using 2 pair guides. *PVT1* expression was slightly decreased after interference with *CASC11* expression. Concomitant *PVT1-CASC11* perturbation in **(E)** LoVo and **(F)** HCT116 cells. CP1: *CASC11-PVT1* sgRNA pair 1, CP2: *CASC11-PVT1* sgRNA pair 2, CP3: *CASC11-PVT1* sgRNA pair 3, and control: non-targeting gRNA. *MYC* expression was increased by interference with *PVT1* and *CASC11* expression. **(G)** Schematic illustration of in cis *CASC11/MYC/PVT1* interaction without and with using CRISPRi system. *P<0.05, **P<0.01, ***P<0.001, ****P<0.0001.

To investigate the effect of *PVT1* expression on *MYC* expression in LoVo and HCT116 colorectal cancer cells, interference with *PVT1* transcription was done using three gRNAs (pair 1, pair 2, and pair 3) *via* the CRISPRi system (**Figures 3A–C**). Three primer sets were used to evaluate the expression of *PVT1* in linear (*linearPVT1*) and circular (*circPVT1*) forms. As shown in **Figure 4A**, applying all gRNAs (P1, P2, and P3) led to a significant reduction in the expression of *circPVT1* in LoVo cells. In contrast, *MYC* and *CASC11* showed an opposing trend of expression, and their expression was increased following *PVT1* knockdown. In HCT116 cells (**Figure 4B**), the expression of *CircPVT1* was decreased by applying all gRNAs, particularly P2. Also, using two sets of primers, 1 and 2, indicated that using P3 significantly knocks down *PVT1* transcripts. The expression of *MYC* and *CASC11* was significantly elevated after *PVT1* knockdown by P1.

In LoVo and HCT116 cells, the expression of *CASC11* was perturbed using two gRNA pairs (C1 and C2) through the CRISPRi system (**Figures 3A–C**). In LoVo cells, the expression of *CASC11* was significantly decreased using C1. However, no significant change was observed in *MYC* expression. The expression of *CircPVT1* and *linear-PVT1* was also significantly reduced. In HCT116 cells, the expression of *CASC11* was decreased but not significantly, the expression of *MYC* did not change significantly, and the expression of *CircPVT1* and *linear-PVT1* was decreased significantly (**Figures 4C, D, G**).

### *PVT1-CASC11* concomitant perturbation causes *MYC* overexpression

The simultaneous use of different combinations of guides targeting *PVT1* and *CASC11* in LoVo cells led to reduced expression of the linear and circular forms of *PVT1*. *CASC11* expression was decreased using two combinations (CP1 and CP2) and increased using one combination (CP3), while *MYC* expression was increased significantly. In HCT116 cells, the expression of the linear and circular forms of *PVT1* was decreased, *CASC11* expression did not change, and *MYC* expression was enhanced significantly (**Figures 4E, F** **)**.

### *PVT1* and *CASC11* perturbation decreases the growth of LoVo cells

**Figure 5:**
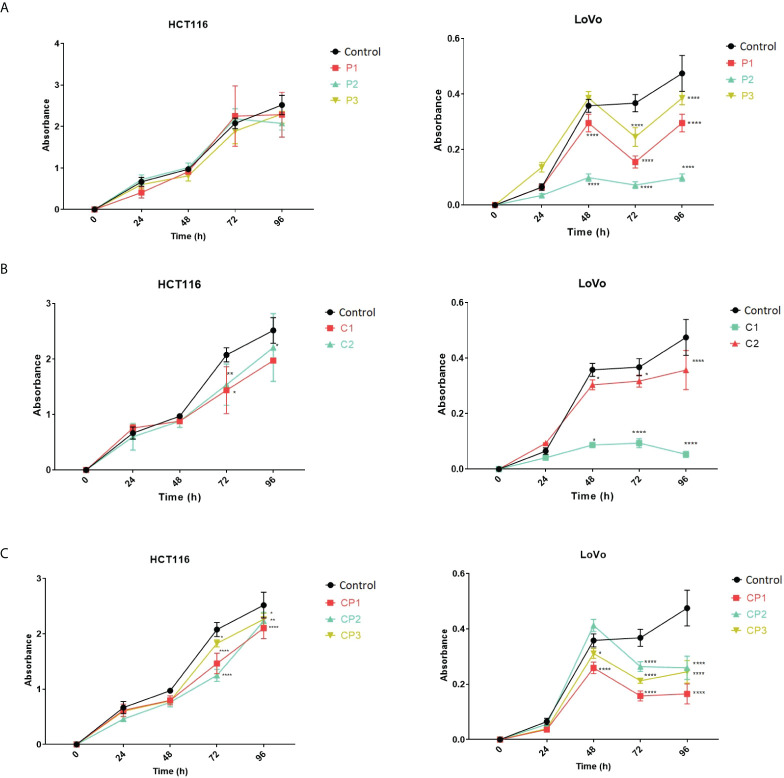
LoVo and HCT116 cell growth in normal and *PVT1* and *CASC11* knocked down forms. **(A)** Cells with *PVT1* expression perturbation, P1: *PVT1* sgRNA pair 1, P2: *PVT1* sgRNA pair 2, and P3: *PVT1* sgRNA pair 3, and control cells with non-targeting gRNA. **(B)** Cells with *CASC11* expression perturbation, C1: *CASC11* sgRNA pair 1, C2: *CASC11* sgRNA pair 2, and control: non-targeting gRNA. **(C)** Concomitant *PVT1-CASC11* perturbation, CP1: *CASC11-PVT1* sgRNA pair 1, CP2: *CASC11-PVT1* sgRNA pair 2, CP3: *CASC11-PVT1* sgRNA pair 3, and control: non-targeting gRNA. Cell growth was significantly decreased 48h after interference with *PVT1* and *CASC11* expression in LoVo cells. *P<0.05, **P<0.01, ****P<0.0001.

To gain insight into the effect of *PVT1* and *CASC11* on the proliferation of LoVo and HCT116 cells, cell growth was assessed using MTT after perturbation of *PVT1* and *CASC11* transcription. In general, a noticeable reduction in cell growth was observed in LoVo cells after perturbation of the expression of *PVT1* and *CASC11*, particularly *via* C1 guides (**Figure 5**). Targeting both *PVT1* and *CASC11* reduced cell growth, particularly in LoVo cells (**Figure 5**).

### *CACS11* is localized mostly in the nucleus, and *PVT* transcripts are localized differently in the nucleus and cytoplasmic parts of LoVo and HCT116 cells

**Figure 6:**
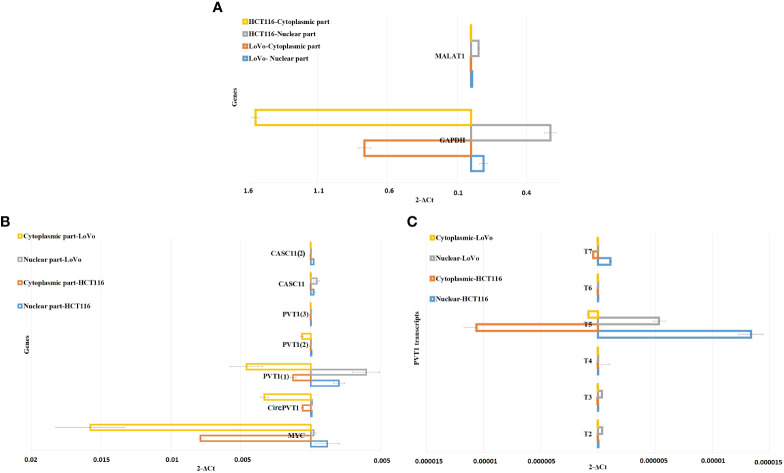
Nuclear/cytoplasmic localization of *MYC*, *PVT1*, and *CASC11* in LoVo and HCT116. **(A)** *MALAT1* and *GAPDH* were used as nuclear/cytoplasmic controls, respectively. **(B)** Nuclear/cytoplasmic localization of *MYC*, *PVT1*, and *CASC11* and **(C)** *PVT1* alternative transcripts in LoVo and HCT116 cells.

To investigate the subcellular localization of *PVT1* and *CACS11*, HCT116 and LoVo cells were fractionated and gene expression analysis was performed for the nuclear and cytoplasmic fractions. We found that *MYC* and *CircPVT1* are localized mostly in the cytoplasmic part, whereas *CASC11* and most of the linear transcripts of *PVT1* are mostly localized in the nuclear part. T4 and T6 did not show any detectable expression in the total RNA of these cells, though they were slightly detectable in cell compartments. Interestingly, T3 was localized preferably in the nucleus of LoVo cells but localized more in the cytoplasmic part in HCT116 cells (**Figure 6**; **Table 1**). The different transcripts of *PVT1* are localized distinctly, which might be explained by RNA element codes or structures produced as a consequence of the particular arrangement of exons.

**Table 1:** Fold changes of *MYC*, *PVT1*, and *CASC11* in the nucleus to the cytoplasm (N/C) and cytoplasm to nucleus (C/N) parts.

Genes	LoVo		HCT116	
	N/C	C/N	N/C	C/N
*GAPDH*		8.43 ± 0.09		2.71 ± 0.25
*MALAT1*	29.38 ± 0.25		39.16 ± 0.51	
*MYC*		75.16 ± 0.90		6.71 ± 0.43
*CircPVT1*		33.43 ± 0.48		9.26 ± 0.08
*PVT1* (1)		1.16 ± 0.11	1.59 ± 0.14	
*PVT1* (2)		32.02 ± 0.41	9.78 ± 0.76	
*PVT1* (3)	2.11 ± 0.22			3.07 ± 0.24
*CASC11* (1)	29.34 ± 0.39		102.60 ± 0.11	
*CASC11* (2)	6.78 ± 0.66		6.15 ± 0.54	
MG562504.1 (T2)	16.52 ± 0.19		2.63 ± 0.23	
MG562505.1 (T3)	11.97 ± 0.21			1.92 ± 0.13
MG562506.1 (T4)	No detectable expression	No detectable expression	Slight detection in the nucleus	No detectable expression
MG562507.1 (T5)	6.40 ± 0.58		1.26 ± 0.08	
MG562508.1 (T6)	No detectable expression	Slight detection in the cytoplasm	No detectable expression	No detectable expression
MG562509.1 (T7)	1.62 ± 0.15		2.67 ± 0.16	

N, nuclear part; C, cytoplasmic part.

Considering the localization of some *PVT1* transcripts in the cytoplasm as well as the high level of their expression, e.g., *CircPVT1* and T5, we assessed their potency for peptide coding using ORFfinder (https://www.ncbi.nlm.nih.gov/orffinder/). ORFfinder results indicated that the longest coding open reading frame (ORF) in *circPVT1* and T5 can encode peptides with 104 and 89 amino acids (**Supplementary Figure S2**).

## Discussion

The MYC oncogene is known to be located on the 8q24 chromosomal site. Adjacent to *MYC*, *PVT1* is located downstream. The gain of function of the 8q24 region, harboring *MYC*, is a frequent variation in various cancers. Although *MYC* is the usual suspect in these malignancies, the role of other co-gained loci remains mostly unknown (31–35). *PVT1* is also a mutational hotspot that is frequently overexpressed in different tumors (35). Recent advancements have led to increasing insights into the critical roles of *PVT1* in cancer initiation and progression. Up to now, a variety of activities have been found for *PVT1*, including modulation of miRNA expression, interaction with proteins, targeting of regulatory genes, formation of fusion genes, functioning as a competing endogenous RNA (ceRNA), and interaction with *MYC* (36). *CASC11*, located upstream of *MYC*, is less known in this region. Recently, it has been reported that *CASC11* might play a role in the carcinogenesis of CRC, gastric cancer, hepatocellular carcinoma (HCC), and cervical cancer (CC) *via* activating Wnt/β-catenin and PI3K/AKT signaling pathways (37).

In the current study, we aimed to evaluate the expression as well as the possible relationship among *MYC*, *PVT1*, and *CASC11* in colorectal cancer. So far, different isoforms of *PVT1* ncRNAs have been annotated, and these splice transcripts could have distinct characteristics in carcinogenesis. With the advancement of transcriptome sequencing, it has been known that splicing is altered broadly in human cancers. Interestingly, oncogenes and tumor suppressor genes are often enriched among the alternatively spliced genes (38, 39). We tried to detect some frequent alternative transcripts of *PVT1* in CRC tissue specimens and assessed their expression levels individually. Our results indicated statistically significant overexpression for T2, T3, and T4 in CRC in comparison with normal margin samples. *PVT1* circular RNA (*circPVT1*), *MYC*, and *CASC11* were significantly overexpressed in these samples. It seems that different transcripts have distinct patterns of expression in CRC. Our findings are consistent with previous studies in which *PVT1* has been identified to be elevated in the CRC tissues (40–43). Some positive correlations found in CRC were moderate (*R*~0.5, *p* < 0.05) (**Supplementary Tables S3, S4**; **Supplementary Figure S1**). Correlation coefficient analyses in tumor and normal samples showed overall increased correlations in tumors compared with normal groups (**Supplementary Figure S1**). It seems that the expression of these transcripts are more correlated in the cancerous state of colorectal cells compared to the normal condition. The similarity in the expression profiles of these genes suggests that they may share a regulatory mechanism of expression or that one of these genes may regulate the expression of the other. Also, positive strong correlation coefficients among some *PVT1* transcripts might be explained by their similar splicing process from longer transcripts (such as T5/T6 and T5/T3), though common TSSs or expression regulatory elements could also play a possible role (such as T4/T6).

We investigated the correlation between gene expression and clinicopathological features in tumor samples. We found that *MYC* expression was significantly correlated with tumor size, and pathological T. MG562507.1 (T5) was significantly correlated with family history. In concordance with our study, some reports have already indicated that *PVT1* expression is associated with clinicopathological characteristics and reduced survival times in CRC patients (44). In addition, other reports have demonstrated that *CASC11* overexpression is associated with tumor-node-metastasis (TNM) and tumor size in CRC (19). *MYC* overexpression in CRC has been shown to be positively associated with age, depth of invasion, lymph node metastasis, and TNM stage (45).

An increasing number of studies provide evidence for the notion that a significant fraction of lncRNAs prefer to stay in the nucleus and participate in epigenetic regulation, nuclear architecture, phase separation, compartment formation, nuclear organization, gene expression, and genomic instability. LncRNAs also regulate cytoplasmic RNAs *via* contributing to mRNA turnover, translation, protein stability, sponging of cytosolic factors, and modulation of signaling pathways (46, 47). We assessed subcellular localization for understudied transcripts in both cell lines, LoVo and HCT116. As expected, *MYC* mRNA is mostly localized in the cytoplasmic part. *CASC11* is localized more in the nuclear rather than the cytoplasmic part. Contrary to our study, a higher expression of *CASC1* has been detected in the cytosolic compared with the nuclear fraction of SW480 and SW620 cells (19). *CircPVT1* is located mostly in the cytoplasmic part. Most linear transcripts of *PVT1* are localized more in the nucleus compared to the cytoplasm. T4 and T6 did not show a significant expression in LoVo and HCT116 cells. Interestingly, T3 is located preferably in the nuclear part of LoVo cells but mostly in the cytoplasmic part of HCT116 cells. In line with our findings, FISH analyses revealed that *PVT1* has a nuclear and cytoplasmic localization in elongated myoblasts (48). Shigeyasu et al. found that the majority of *PVT1* lncRNA is found within the nuclear compartment of HCT116 cells (49). The presence of *PVT1* circular and linear transcripts in both nuclear and cytoplasmic parts could provide a tendency for a variety of functions in cells, while the lower expression level of *CASC11* and its localization in the nucleus highlight the nuclear-related roles of this lncRNA in LoVo and HCT116 cells.

In general, lncRNAs are poorly spliced (50, 51), and their splicing has been linked to increased enhancer activity (52). Some enhancer elements with conserved splicing signals serve as promoters for the production of lncRNA transcripts (53). The splicing of RNA transcripts, both coding and noncoding, has been revealed to cause the increased expression of nearby genes (54, 55) Shigeyasu et al. have reported that the *PVT1* locus with significantly high enhancer activity in CRC can regulate the expression of the *MYC* oncogene (49). To investigate the ability of *PVT1* and *CASC11* lncRNAs to affect the expression of *MYC* in CRC cells, we performed knockdown experiments for these lncRNAs using CRISPRi. We found that perturbation of *PVT1* expression causes *MYC* overexpression in LoVo and HCT116 cells. *CASC11* expression perturbation caused a slight decrease in *PVT1* expression and did not have a significant impact on *MYC* expression. Based on these observations, it seems that *PVT1* expression and possibly the euchromatin state of the *PVT1* transcription start site could negatively control *MYC* expression, especially in LoVo cells. Our results are in line with the study of Cho et al., which showed *PVT1* and *MYC* promoters compete for enhancer contact in *cis* in breast cancer cell lines. However, in contrast to our findings, they suggested that this interaction occurs in a cell type-specific manner, PVT1-CRISPRi cannot induce *MYC* expression in HCT116 cells, and *MYC* loops to the *CCAT1* enhancer instead of *PVT1* in these cells (56). However, Shigeyasu et al. showed that the *PVT1* locus with enhancer activity forms a loop structure in a *cis* conformation with *MYC* in HCT116 cells (49) that can explain our findings. The use of dCAS9/KRAB can turn chromatin to heterochromatin state. As a result, the DNA region containing *PVT1* transcription start site cannot easily communicate with the enhancer area in its vicinity. Therefore, it seems that while *PVT1* RNAs have an oncogenic role, *PVT1* gene regulatory DNA can have a tumor-suppressive role. We also assessed *MYC* expression with simultaneous perturbation in both *PVT1* and *CASC11* transcription. Concomitant *PVT1-CASC11* perturbation caused *MYC* overexpression in LoVo and HCT116. Consistent with the potent growth-promoting properties of Myc, cells have evolved a plethora of mechanisms to regulate their expression and activity (57). It seems that *MYC* is embedded in loci with regulatory DNA and RNA elements, which can control its expression *in situ* at the transcription level. These recent findings, along with our study, highlight the fact that more detailed information is required to shed light on the exact role of *PVT1* and other similar ncRNA genes harboring regulatory DNA elements, and our current knowledge only covers the tip of the iceberg.

Our observations indicated that *CASC11* and *PVT1* expression perturbation decreases LoVo and HCT116 cell growth. This might provide some evidence for the MYC-independent role of lncRNAs in the proliferation of CRC cells. This finding is in concordance with previous studies showing that *CASC11* can play a role in CRC progression *via* activation of the WNT/β-catenin pathway and *PVT1* by regulating the MiR-106b-5p/FJX1 axis, and knockdown of *PVT1* or *CASC11* suppresses the proliferation of CRC cells (19, 40). It is noteworthy that C1 guides remarkably decreased LoVo cell growth. This pair of RNA guides targets the *CASC11* transcription start site, overlapping with a replication origin-like site 5′ to the *MYC*, and showed autonomous replicating sequence activities (58). A decrease in LoVo cell growth could be in part caused by the interference of CRISPRi with the replication-like origin region upstream of *MYC*. On the other hand, MYC upregulation triggers cell-autonomous apoptosis in normal tissues through p53-related pathways (59, 60). We assumed that cell growth might decrease upon *MYC* overexpression followed by *PVT1*-*CASC11* perturbation, in part, due to the existence of p53 wild type in both HCT116 and LoVo cells. Further *PVT1*-*CASC11* CRISPRi experiments in p53-null CRC cell lines such as Caco-2 can confirm this initial hypothesis. Taken together, our results provide convincing evidence for the notion that noncoding genes, *PVT1* and *CASC11*, located in 8q24 play important roles in the carcinogenesis of CRC tumors. Our data highlight that these noncoding genes might serve as biomarkers for diagnosis and potential therapeutic targets in CRC patients.

## Data availability statement

The datasets presented in this study can be found in online repositories. The names of the repository/repositories and accession number(s) can be found in the article/**Supplementary Material**.

## Ethics statement

The studies involving human participants were reviewed and approved by Ethics Committee of Shahid Chamran University of Ahvaz, Ahvaz, Iran. The patients/participants provided their written informed consent to participate in this study.

## Author contributions

MZ, HG, and RJ contributed to the conception and design of the study. MZ performed the experiments. MZ performed the experiments. MZ wrote the first draft of the manuscript with the support of BB. HG, RJ, A-MF, and M-RH supervised the work. All authors discussed the results, contributed to manuscript revision, and read and approved the submitted version.

## Acknowledgments

We acknowledge the Faculty of Science, Shahid Chamran University of Ahvaz, Ahvaz, Iran for supporting us in this project. We wish to thank all the staff of the Narges Medical Genetics and Prenatal Diagnosis Laboratory (Ahvaz, Iran) and all the gold lab members (Johnson’s lab, DBMR, University of Bern, Bern, Switzerland) for their experimental support.

## Conflict of interest

The authors declare that the research was conducted in the absence of any commercial or financial relationships that could be construed as a potential conflict of interest.

## Publisher’s note

All claims expressed in this article are solely those of the authors and do not necessarily represent those of their affiliated organizations, or those of the publisher, the editors and the reviewers. Any product that may be evaluated in this article, or claim that may be made by its manufacturer, is not guaranteed or endorsed by the publisher.

## Abbreviations

*MYC*, myelocytomatosis; *PVT1*, plasmacytoma variant translocation 1; *CASC11*, cancer susceptibility candidate 11; *ACTB*, Beta-actin; *GAPDH*, glyceraldehyde 3-phosphate dehydrogenase; *MALAT1*, metastasis-associated lung adenocarcinoma transcript 1; CRC, colorectal cancer; lncRNA, long noncoding RNA; circRNA, circular RNA; SNV, single nucleotide variations; CNV, copy number variation; CRISPR, clustered regularly interspaced short palindromic repeats; CRISPRi, CRISPR interference; DMEM, Dulbecco’s modified Eagle’s medium; FBS, fetal bovine serum; Q-PCR, quantitative PCR; FACS, fluorescence-activated cell sorting; RFP, red fluorescent protein; MTT, 3-(4,5-dimethylthiazol-2-yl)-2,5-diphenyl-2H-tetrazolium bromide; ROC, receiver operating characteristic; HCC, hepatocellular carcinoma; CC, cervical cancer.
