# Supplementary material for: CASC11 and PVT1 spliced transcripts play an oncogenic role in colorectal carcinogenesis
Source: Front Oncol. 2022 Aug 16;12:954634. doi: 10.3389/fonc.2022.954634 (PMC9424822; doi:10.3389/fonc.2022.954634)
Supplement: Supplementary file 1 [file DataSheet_1.docx]

**Supplementary Table 1** Primers used for q-PCR

| Amplicon (bp) | Sequence | Primer | RefSeq (assembly: GRCh38.p13) | Gene/ transcript |
| --- | --- | --- | --- | --- |
| 321 | F: TGGGCTTGAGCTGACCATACTC | F12 R46 | MG562504.1 | T2 |
|  | R: TTATTCTGTCCTTCTTAATTCTCCAATC |  |  |  |
| 153 | F: CTGGGCTTGAGAATCCTGTTAC | F14 R46 | MG562505.1 | T3 |
|  | R: TTATTCTGTCCTTCTTAATTCTCCAATC |  |  |  |
| 269 | F: GAGATTTGGAGAGAATCCTGTTACACC | F24 R56 | MG562506.1 | T4 |
|  | R: TTATTCTGTCCTTCTCATGTCCACTAG |  |  |  |
| 152 | F: TGAGCTGAGCAGTTAATGCTAGG | FN_1_N_2_ RN_2_6 | MG562507.1 | T5 |
|  | R: TTATTCTGTCCTTCACCAAAATCCC |  |  |  |
| 265 | F: TGAGCTGAGCAGTTAATGCTAGG | FN_1_N_2_ R56 | MG562508.1 | T6 |
|  | R: TTATTCTGTCCTTCTCATGTCCACTAG |  |  |  |
| 158 | F: GAGATTTGGAGAGAATCCTGTTACACC | F24 R48 | MG562509.1 | T7 |
|  | R: AGACATGCAAAAAACCTTAATTCTCCAATC |  |  |  |
| 144 | F: CGACTCTTCCTGGTGAAGCATCTGAT | Circ-PVT1-F,R | - | CircPVT1 |
|  | R: TACTTGAACGAAGCTCCATGCAGC |  |  |  |
| 86 | F: CTTGGAGGCTGAGGAGTTCA | PVT1-F,R | [NR_003367.3](https://www.ncbi.nlm.nih.gov/entrez/viewer.fcgi?db=nucleotide&id=929524279) | PVT1 |
|  | R: CTTCAGGCCTCTTTGACAGC |  |  |  |
| 158 | F: CCGGCACATTTCAGGATAC | PVT1 (2)-F,R | [NR_003367.3](https://www.ncbi.nlm.nih.gov/entrez/viewer.fcgi?db=nucleotide&id=929524279) | PVT1 (2) |
|  | R: CCAAAGGAAGTTGGCAGAG |  |  |  |
| 95 | F: GAAGCAATTCAGCCCAACAG | PVT1 (3)-F,R | [NR_003367.3](https://www.ncbi.nlm.nih.gov/entrez/viewer.fcgi?db=nucleotide&id=929524279) | PVT1 (3) |
|  | R: GGTGGACAGGTAACAGGTG |  |  |  |
| 183 | F: GCGACTCTGAGGAGGAAC | MYC-F,R | [NM_002467.6](https://www.ncbi.nlm.nih.gov/entrez/viewer.fcgi?db=nucleotide&id=1552482295)  [NM_001354870.1](https://www.ncbi.nlm.nih.gov/entrez/viewer.fcgi?db=nucleotide&id=1236774668) | MYC |
|  | R: CTGCGTAGTTGTGCTGATG |  |  |  |
| 110 | F: GCAGAAGGTCCGAAGAAAGAG | CASC11-F,R | [NR_117102.1](https://www.ncbi.nlm.nih.gov/entrez/viewer.fcgi?db=nucleotide&id=620597373)  [NR_117101.1](https://www.ncbi.nlm.nih.gov/entrez/viewer.fcgi?db=nucleotide&id=620597381) | CASC11 |
|  | R: TGTTCATTAGCAGTGGTGATAGG |  |  |  |
| 91 | F: ATTGGCAATGAGCGGTTC | ACTB-F,R | [NM_001101.5](https://www.ncbi.nlm.nih.gov/entrez/viewer.fcgi?db=nucleotide&id=1519311456) | ACTB |
|  | R: TGAAGGTAGTTTCGTGGATG |  |  |  |
| 150 | F: GATTGAGGCGTTTTCCAAGA | MALAT1-F,R | [NR_144568.1](https://www.ncbi.nlm.nih.gov/entrez/viewer.fcgi?db=nucleotide&id=1108847773)  [NR_144567.1](https://www.ncbi.nlm.nih.gov/entrez/viewer.fcgi?db=nucleotide&id=1108847772)  [NR_002819.4](https://www.ncbi.nlm.nih.gov/entrez/viewer.fcgi?db=nucleotide&id=1108618392) | MALAT1 |
|  | R: ACTTTCTCCCCCAACTGCTT |  |  |  |
| 138 | F: GCACCGTCAAGGCTGAGAAC | GAPDH-F,R | [NM_001256799.3](https://www.ncbi.nlm.nih.gov/nuccore/NM_001256799.3)  [NM_001289745.3](https://www.ncbi.nlm.nih.gov/nuccore/NM_001289745.3)  [NM_001289746.2](https://www.ncbi.nlm.nih.gov/nuccore/NM_001289746.2)  [NM_001357943.2](https://www.ncbi.nlm.nih.gov/nuccore/NM_001357943.2)  [NM_002046.7](https://www.ncbi.nlm.nih.gov/nuccore/NM_002046.7) | GAPDH |
|  | R: TGGTGAAGACGCCAGTGGA |  |  |  |

**Supplementary Table 2** sgRNA positions and sequences used for CRISPRi.

| Guide sequence | Sequence |  | Distance from TSS (+1) |
| --- | --- | --- | --- |
| PVT1 pair 1 | **GTCGTCGCCCCTCCTCGTCC** | TSS1  TTS2 | 53-73 |
|  | **AGTGGTCTGGGGAATAACGC** |  | 69-89 |
| PVT1 pair 2 | **CGGGGCGGCCGGGACGAGGA** | TSS1 TTS2 | 56-76 |
|  | **TACAGTGATCTTCAGTGGTC** |  | 56-76 |
| PVT1 pair 3 | **CGGCCGAGCACATGGGCCCG** | TSS1 | 21-41 |
|  | **CGAGAGTTTCCAGAAACGCA** |  | 223-243 |
| CASC11 pair 1 | **GAAGAAAGAGGAGTTACTGG** | TSS1 | 32-52 |
|  | **ACCCTATGGAGAACCGGTAA** |  | 147-167 |
| CASC11 pair 2 | **TGGAGGAAAAAGTGGTTCAG** | TSS1 | 49-69 |
|  | **AAAATTTGAAAGCAAGACAC** |  | 275-295 |

TSS: Transcription start site

**Supplementary Table 3** Spearman correlation coefficients of gene expression in CRC samples (tumor to normal).

| **CRC (T/N)**  **SPEARMAN R** | **MYC** | **CASC11** | **PVT1** | **T2** | **T3** | **T4** | **T5** | **T6** | **T7** | **PVT1Circ** |
| --- | --- | --- | --- | --- | --- | --- | --- | --- | --- | --- |
| **MYC** |  | R=0.085  p−value =0.70 | R=0.325  p−value =0.20 | R=0.167  p−value =0.44 | R=0.079  p−value =0.71 | R=-0.055  p−value =0.81 | R=0.041  p−value =0.85 | R=-0.286  p−value =0.19 | R=0.262  p−value =0.22 | R=0.093  p−value =0.66 |
| **CASC11** |  |  | R=-0.012 p−value =0.96 | R=0.148 p−value =0.46 | R=0.542 p−value =0.003 | R=0.182 p−value =0.42 | R=0.39  p−value =0.04 | R=0.052  p−value =0.8 | R=0.179  p−value =0.4 | R=0.139  p−value =0.48 |
| **PVT1** |  |  |  | R=0.214 p−value =0.44 | R=-0.170 p−value =0.5 | R=-0.35 p−value =0.18 | R=-0.042 p−value =0.87 | R=0.046  p−value =0.86 | R=0.0423  p−value =0.87 | R=0.187  p−value =0.44 |
| **T2** |  |  |  |  | R=-0.031 p−value =0.87 | R=0.092 p−value =0.67 | R=0.200 p−value =0.31 | R=0.196  p−value =0.33 | R=0.134  p−value =0.49 | R=-0.148  p−value =0.45 |
| **T3** |  |  |  |  |  | R=0.313 p−value =0.14 | R=0.472 p−value =0.01 | R=0.264  p−value =0.18 | R=0.349  p−value =0.07 | R=0.305  p−value =0.11 |
| **T4** |  |  |  |  |  |  | R=0.450 p−value =0.03 | R=0.450  p−value =0.03 | R=0.0178  p−value =0.94 | R=-0.118  p−value =0.61 |
| **T5** |  |  |  |  |  |  |  | R=0.449  p−value =0.02 | R=0.541  p−value =0.003 | R=0.117  p−value =0.55 |
| **T6** |  |  |  |  |  |  |  |  | R=0.135  p−value =0.50 | R=0.075  p−value =0.71 |
| **T7** |  |  |  |  |  |  |  |  |  | R=0.124  p−value =0.53 |
| **CircPVT1** |  |  |  |  |  |  |  |  |  |  |

Significant data (p<0.05) are underlined

**Supplementary Table 4** Spearman correlation coefficients of gene expression and clinicopathological characteristics of CRC samples (tumor to normal).

| **CRC (T/N) SPEARMAN R** | **Tumor size (cm)** | **Histology** | **Histology grade** | **Lymphatic invasion** | **Vascular invasion** | **Perineural invasion** | **Perineal Invasion** | **Extracaps. Nodal Extension** | **Perforation** | **Peritoneal Seeding** | **Pathological T** | **Pathological N** | **Clinical Metastasis** | **TNM staging** | **Family history** | **Smoking status** | **Alcohol consumption** | **Weithloss (kg)** |
| --- | --- | --- | --- | --- | --- | --- | --- | --- | --- | --- | --- | --- | --- | --- | --- | --- | --- | --- |
| **MYC** | R=0.434  p=0.034 | R=0.175  p=0.411 | R=0.316  p=0.132 | R=-0.225  p=0.289 | R=-0.225  p=0.289 | R=-0.247  p=0.245 | R=0.106  p=0.621 | R=0.230  p=0.279 | R=-0.167  p=0.435 | R=0.040  p=0.853 | R=0.602  p=0.00.2 | R=-0.016  p=0.94 | R=0.288  p=0.172 | R=0.252  p=0.235 | R=0.120  p=0. 580 | R=0.335  p=0.110 | R=0.335  p=0.110 | R=-0.264  p=0.233 |
| **CASC11** | R=-0.121  p=0.550 | R=0.201  p=0.314 | R=-0.333  p=0.090 | R=-0.068  p=0.735 | R=-0.068  p=0.735 | R=0.084  p=0.677 | R=-0.203  p=0.310 | R=0.107  p=0.600 | R=.101  p=0.614 | R=-0.108  p=0.590 | R=0.026  p=0.900 | R=0.186  p=0.351 | R=0.330  p=0.092 | R=0.277  p=0.162 | R=-0.198  p=0.323 | R=-0.312  p=0.120 | R=-0.226  p=0.267 | R=-0.292  p=0.176 |
| **PVT** | R=-0.058  p=0.813 | R=-0.094  p=0.702 | R=-0.028  p=0.910 | R=0.219  p=0.367 | R=0.219  p=0.367 | R=0.262  p=0.280 | R=-0.215  p=0.380 | R=0.438  p=0.060 | R=0  p=1 | R=0.400  p=0.090 | R=0  p=1 | R=-0.503  p=0.030 | R=0.258  p=0.286 | R=-0.376  p=0.112 | R=0.011  p=0.964 | R=-0.061  p=0.802 | R=0.053  p=0.828 | R=0.193  p=0.443 |
| **T2** | R=-0.152  p=0.458 | R=-0.096  p=0.640 | R=-0.160  p=0.435 | R=0.148  p=0.470 | R=0.148  p=0.470 | R=-0.160  p=0.446 | R=-0.070  p=0.745 | R=-0.024  p=0.907 | R=0.174  p=0.395 | R=-0.062  p=0.762 | R=-0.137  p=0.500 | R=0.016  p=0.937 | R=-0.280  p=0.164 | R=-0.05  p=0.809 | R=-0.07  p=0.741 | R=-0.06  p=0.776 | R=-0.213  p=0.307 | R=0.099  p=0.660 |
| **T3** | R=0.102  p=0.606 | R=0.017  p=0.930 | R=-0.030  p=0.877 | R=0.178  p=0.388 | R=0.170  p=0.388 | R=0.270  p=0.166 | R=-0.300  p=0.124 | R=0.350  p=068 | R=-0.083  p=0.673 | R=-0.095  p=0.628 | R=0.038  p=0.848 | R=0.006  p=0.975 | R=0.274  p=0.158 | R=0.201  p=0.304 | R=-0.317  p=0.100 | R=-0.218  p=0.274 | R=-0.189  p=0.344 | R=-0.079  p=0.713 |
| **T4** | R=0.016  p=0.940 | R=0.279  p=0.197 | R=-0.509  p=0.013 | -0.223  p=0.306 | R=-0.223  p=0.306 | R=0.298  p=0.166 | R=0.064  p=0.770 | R=0.093  p=0.673 | R=0.257  p=0.236 | R=-0.149  p=0.498 | R=0.005  p=0.981 | R=-0.101  p=0.646 | R=0.289  p=0.180 | R=0.099  p=0.654 | R=-0.345  p=0.106 | R=-0.270  p=0.223 | R=-0.253  p=0.256 | R=-0.015  p=0.948 |
| **T5** | R=0.250  p=0.199 | R=0  p=1 | R=-0.169  p=0.390 | 0.080  p=0.684 | R=0.080  p=0.684 | R=0.117  p=0.551 | R=0.036  p=0.857 | R=0.336  p=0.080 | R=0.274  p=0.158 | 0.108  p=0.582 | R=-0.012  p=0.950 | R=-0.128  p=0.515 | R=0.226  p=0.247 | R=0.1  p=0.613 | R=-0.386  p=0.043 | R=-0.286  p=0.148 | R=-0.299  p=0.129 | R=0.109  p=0.613 |
| **T6** | R=-0.093  p=0.645 | R=-0.31  p=0.117 | R=-0.266  p=0.180 | R=0.191  p=0.339 | R=0.191  p=0.339 | R=0.195  p=0.330 | R=-0.277  p=0.162 | R=0.030  p=0.880 | R=.252  p=0.205 | R=0.194  p=0.331 | R=-0.306  p=0.119 | 0.043  p=0.829 | R=-0.327  p=0.095 | R=-0.112  p=0.578 | R=-0.324  p=0.099 | R=-0.110  p=0.591 | R=-0.272  p=0.178 | R=0.175  p=0.424 |
| **T7** | R=0.227  p=0.246 | R=0.051  p=0.795 | R=-0.134  p=0.497 | R=0.366  p=0.055 | R=0.366  p=0.055 | R=0.029  p=0.882 | R=-0.155  p=0.431 | R=-0.007  p=0.971 | R=-0.107  p=0.587 | R=0.027  p=0.891 | R=0.256  p=0.187 | R=-0.304  p=0.116 | R=0.3  p=0.124 | R=0.03  p=0.558 | R=-0.220  p=0.259 | R=-0.152  p=0.448 | R=-0.066  p=0.743 | R=0.175  p=0.412 |
| **PVT1**  **Circ**  **PVT1** | R=0.300  p=0.120 | R=0.02  p=0.931 | R=0.3  p=0.148 | R=0.05  p=0.804 | R=0.05  p=0.804 | R=0.1  p=0.638 | R=0.036  p=0.866 | R=0.23  p=0.241 | R=-0.083  p=0.673 | R=0.148  p=0.451 | R=0.210  p=0.282 | R=0.16  p=0.422 | R=0.13  p=0.506 | R=0.31  p=0.111 | R=-0.022  p=0.911 | R=-0.022  p=0.915 | R=-0.022  p=0.913 | R=-0.16  p=0.463 |

p indicates p-value.

Significant data (p<0.05) are underlined.


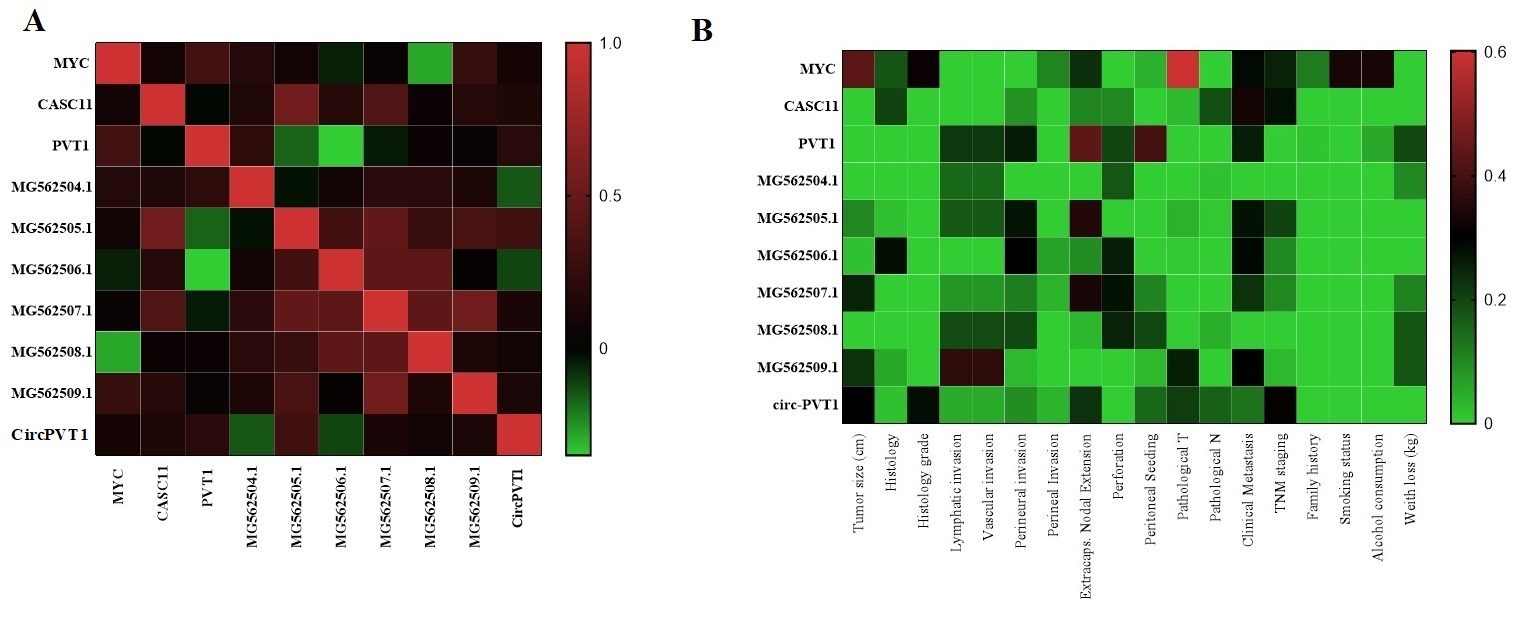


**Supplementary Figure 1** Heat map of A) genes expression correlation and B) gene expression/clinicopathological features correlation.

*
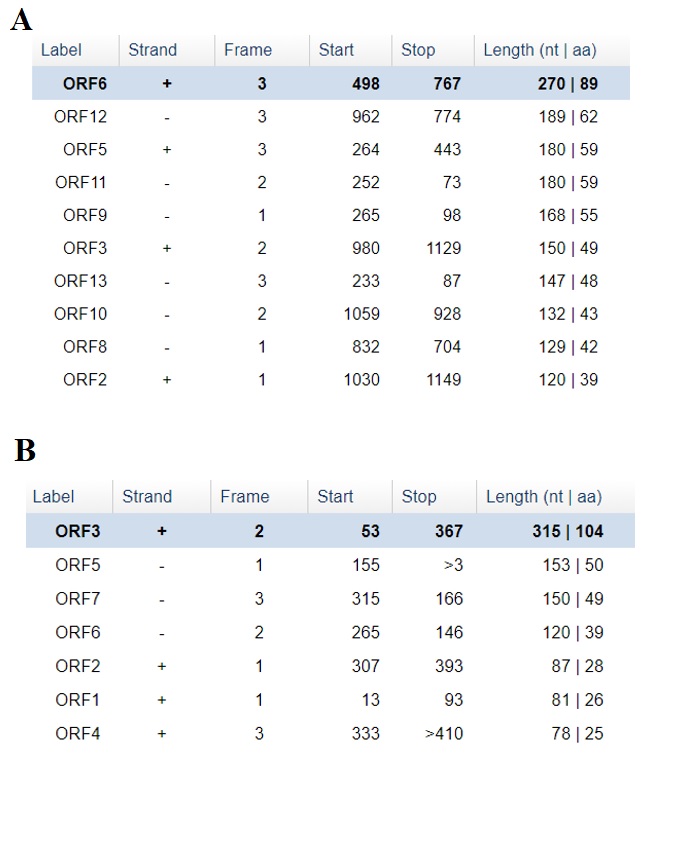
*

**Supplementary Figure 2** ORF search result for T5 (A) and CircPVT1 (B).
